# Economic burden of malaria on businesses in Ghana: a case for private sector investment in malaria control

**DOI:** 10.1186/s12936-016-1506-0

**Published:** 2016-09-06

**Authors:** Justice Nonvignon, Genevieve Cecilia Aryeetey, Keziah L. Malm, Samuel Agyei Agyemang, Vivian N. A. Aubyn, Nana Yaw Peprah, Constance N. Bart-Plange, Moses Aikins

**Affiliations:** 1 Department of Health Policy, Planning and Management, College of Health Sciences, School of Public Health, University of Ghana, Legon, Accra, Ghana; 2 National Malaria Control Programme, Ghana Health Service, Accra, Ghana

**Keywords:** Malaria control, Private sector investment, Economic cost, Ghana

## Abstract

**Background:**

Despite the significant gains made globally in reducing the burden of malaria, the disease remains a major public health challenge, especially in sub-Saharan Africa (SSA) including Ghana. There is a significant gap in financing malaria control globally. The private sector could become a significant source of financing malaria control. To get the private sector to appreciate the need to invest in malaria control, it is important to provide evidence of the economic burden of malaria on businesses. The objective of this study, therefore, was to estimate the economic burden on malaria on businesses in Ghana, so as to stimulate the sector’s investment in malaria control.

**Methods:**

Data covering 2012–2014 were collected from 62 businesses sampled from Greater Accra, Ashanti and Western Regions of Ghana, which have the highest concentration of businesses in the country. Data on the cost of businesses’ spending on treatment and prevention of malaria in staff and their dependants as well as staff absenteeism due to malaria and expenditure on other health-related activities were collected. Views of business leaders on the effect of malaria on their businesses were also compiled. The analysis was extrapolated to cover 5828 businesses across the country.

**Results:**

The results show that businesses in Ghana lost about US$6.58 million to malaria in 2014, 90 % of which were direct costs. A total of 3913 workdays were lost due to malaria in firms in the study sample during the period 2012–2014. Businesses in the study sample spent an average of 0.5 % of the annual corporate returns on treatment of malaria in employees and their dependants, 0.3 % on malaria prevention, and 0.5 % on other health-related corporate social responsibilities. Again business leaders affirmed that malaria affects their businesses’ efficiency, employee attendance and productivity and expenses. Finally, about 93 % of business leaders expressed the need private sector investment in malaria control.

**Conclusions:**

The economic burden of malaria on businesses in Ghana cannot be underestimated. This, together with business leaders’ acknowledgement that it is important for private sector investment in malaria control, provides motivation for engagement of the private sector in financing malaria control activities.

## Background

Globally, there has been a notable reduction in the burden of malaria as evidenced by 2015 estimates compared to 2000 estimates. For instance, the total number of cases, incidence and deaths have reduced by 18, 37 and 48 %, respectively [1]. The mortality rate has also reduced by 60 % over the same period. Within sub-Saharan Africa (SSA), deaths in children under-five years reduced by 57.9 %, displacing malaria to fourth place in terms of cause of deaths. Despite these significant gains, malaria remains a major public health challenge in SSA—about 88 % of the total global cases and 90 % of deaths in 2015 occurred in the region [1]. In 2015, about 10 % of under-five deaths in SSA could be attributed to malaria [1].

In Ghana, malaria accounted for about 38 % of outpatient visits and 27.3 % of admissions in health facilities and 48.5 % of under-five deaths in 2015, remaining one of the leading causes of morbidity and mortality [2]. Ghana and nine other countries in SSA accounted for more than 60 % of malaria deaths in SSA in 2012 [3].

Studies have shown that malaria imposes significant burden on the economy of developing countries [4–7]. Gallup and Sachs [8] estimated the economic burden of malaria on African countries to be up to US$12 billion annually and that between 1965 and 1990, economic growth in developing countries reduced by 1.3 % per person per year due to malaria, whereas McCarthy et al. [9] put the reduction in economic growth due to malaria at 0.25 % [9]. Other estimates show that gross domestic product (GDP) of many developing countries could reduce by 5–6 % due to malaria [10].

Studies have argued that the economic benefits significantly reducing or eliminating malaria are enormous. Purdy et al. argue that if appropriate investments are put into malaria control globally, the net present value of benefits (over costs) that would accrue by 2035 could be US$208.6 billion [11]. The World Health Organization (WHO) estimates that although costs of investing in malaria elimination between 2016–2030 could reach US$101.8 billion (with another US$673 million invested in research and development annually), the returns on such investment could be 40:1 globally and 60:1 for SSA, implying, for example, that for every US$1 spent, economic gains of US$40 or US$60 would be accrue [12]. Similarly, other estimates show that about US$90–US$120 billion are required to eradicate malaria be by 2040, and investments to reduce 50 % of the malaria burden globally could generate economic returns of 36:1 [13]. In terms of the impact of malaria on individuals, Bleakley estimate that malaria infection during childhood reduced adult incomes by about 50 %. Further, studies have shown that a 10 percentage point reduction in malaria incidence could raise literacy 1–2 percentage points [14] and 2.5–5.6 percentage points [15].

Malaria is known to affect businesses in many ways; reduced productivity due to increased worker absenteeism, increased health care spending, which all impact business returns and tax revenue to the state [7, 16]. The 2011 Roll Back Malaria Report indicates further that in SSA, 72 % of businesses reported a negative malaria impact, with 39 % perceiving these impacts to be serious. The report also indicated nearly 75 % of businesses in the Africa region reported that malaria was negatively affecting their business in a survey conducted in 2004.

In Ghana, past estimates of the economic burden of malaria on households and the economy abound. Asante and Asenso-Okyere [17] estimated that a 1 % increase in malaria morbidity reduces economic growth by about 0.41 %, and that an episode of malaria costs households US$15.79 (in 2003 dollars). Abotsi [18] also estimated that an episode of malaria costs households between US$10.20 (uncomplicated malaria) and US$46.62 (severe malaria) (in 2007 dollars). Furthermore, Sicuri et al. [19] found that households spent between US$5.70 (uncomplicated malaria) and US$48.73 (severe) in Ghana.

Current estimates of the economic burden of malaria on businesses in SSA in general, and Ghana in particular, are limited. In 2004, AngloGold Ashanti (a mining company in Ghana) incurred up to $55,000 per month on treatment of malaria in its employees and their dependants [20]. Further, 30 % of business leaders who responded to a survey in Ghana reported that malaria had a strong impact on productivity [21]. The cost estimates by AngloGold Ashanti, nonetheless, represents only direct company expenses on treatment and not the valued productive losses the business incurs due to absenteeism.

The purpose of this study, therefore, is to estimate the economic burden of malaria on businesses in Ghana. Such clear estimates are essential as advocacy tools to stimulate private sector involvement in malaria control in the country, especially as donor funds are reducing due to the country’s current status as a lower-middle income country. In addition, the estimates could be used to create awareness and sensitize businesses that it is economically beneficial to invest in malaria control.

## Methods

### Study design

The study was a cross-sectional study employing quantitative methods to collect expenditure and other cost related data from selected businesses and interviews to elicit views from senior managers.

### Study sites

The study was conducted in three out of the ten regions of Ghana; Ashanti, Greater Accra and Western regions. Available business statistics indicate that these regions have the largest number of businesses with variation in types and sizes of businesses in the country. Further, malaria prevalence in the study regions are 39 % (the third highest prevalence) in Western Region, 16.6 % (third lowest) in Ashanti Region and 11.2 % (the lowest) in Greater Accra Region. Thus, in terms of the burden of malaria, the study regions are spread across the distribution [22].

### Study population

The study population comprises of all businesses within the selected regions registered with the Association of Ghana Industries (AGI) and the Ghana Employers Association (GEA), the two largest association of businesses in the country

### Sample and sampling procedure

The sample size was purposively determined in consultation with the National Malaria Control Programme. It was anticipated that 15–20 businesses per region would suffice to estimate the burden of malaria on businesses. Names of businesses were obtained from Association of Ghana Industries (AGI) and Ghana Employers Association (GEA) Members’ catalogue. Ashanti, Greater Accra and Western regions were purposively selected due to the high concentration of businesses in these three regions. The businesses in each region were then listed and sorted alphabetically by business sectors in Microsoft Excel. The businesses were then purposively selected and grouped into agriculture and agribusinesses, financial, manufacturing and processing and services according to categorization of businesses in the economy. The number of businesses that responded within the data collection period by region were as follows: Ashanti (n = 23), Greater Accra (n = 16) and Western (n = 23) out of a total of 909 businesses registered with the two business associations in Ghana.

### Cost data collection

Data were collected in June/July 2015. Semi-structured questionnaires were used for data collection. The data collected covered the period from 2012 to 2014. Two types of questionnaire were designed for data collection. The first was to obtain information for estimating direct and indirect cost of malaria on businesses. These covered: (1) general information on businesses; (2) businesses’ income and volume of production; (3) direct cost of illnesses (malaria and others); and (4) indirect cost of illnesses (malaria and others). The second questionnaire covered other effects of malaria illness on businesses that could not be directly quantified. These were administered to Chief Executive Officer/Director, Finance Manager, Operations Manager and Human Resource Manager through interviews. Research Assistants vetted the filled questionnaires for completeness.

### Data analysis

The analysis focused on direct costs, indirect cost and other effects of malaria. The direct cost comprised of expenses incurred by the business on: (a) all direct treatment cost of reported illness among staff and their dependants; (b) the direct cost of prevention and treatment of malaria (i.e. employees and their dependants); and (c) the proportion of direct cost of all illnesses due to malaria. The indirect cost (i.e. productivity losses) comprised of: (a) total reported days of absenteeism due to ill-health; (b) reported absenteeism due to malaria; (c) proportion of reported absenteeism due to malaria; (d) estimated productivity loss of the business due to ill-health; (e) estimated productivity loss of the business due to malaria; and (f) estimated reduction in productivity of the business due to malaria. Productivity costs were estimated by multiplying the number of work days lost (i.e. absenteeism due to malaria) by the national minimum daily wage rate. Absenteeism due to malaria was calculated using data from firms who had records on how many days their workers lost due to malaria. For the firms who had no records, estimates on average absenteeism due to malaria out of total absenteeism due to ill-health we used estimates from the NMCP and other studies were used to estimate the total work days lost due to malaria.

These costs and work days lost were systematically compiled under appropriate cost categories and the total costs, average costs and proportions estimated. Based on these sample costs, a national ill-health and malaria cost to businesses was estimated as the average ill-health and malaria costs to business multiplied by the total number of businesses (5828 in all) in the country. Where data were not available for specific business types, average numbers and costs for the given sector were applied.

The other burden (not quantified) of malaria morbidity on businesses in terms of its impact on business operations, views on investments in malaria control, and business expenditure on malaria control activities were grouped into emerging themes and tabulated according to business sectors.

The total cost of malaria to businesses was obtained as a summation of the direct expenditure on malaria and the valued loss productivity costs. The other effects were described as these could not be quantified. All costs were measured in the appropriate year and adjusted into 2014 GHS using consumer price indices for health goods for the respective years. Then, GHS values were converted into 2014 US$ using an exchange rate of US$1 to GHS2.26.

## Results

### Background of study businesses

In terms of size, the businesses included in the study sample had a total workforce of 8141 of which about 69 % were males (Table 1). About 41 % of the employees were in businesses in the Greater Accra Region, 25 % in Ashanti Region and 34 % in Western Region. In terms of business sectors, the services sector accounted for 47 % of all employees with the financial services sector accounting for 0.7 %.

**Table 1:** Background characteristics of study businesses

Indicator	N	%
Total no. of employees	8141	100
No. of employees by gender		
Male	5644	69.3
Female	2497	30.7
No. of employees by sectors		
Construction and engineering	420	5.2
Services	3825	47.0
Manufacturing and processing	1335	16.4
Financial services	57	0.7
Pharmaceutical	537	6.6
Agriculture and agribusiness	1967	24.2
No. of employees by region		
Greater Accra	3304	40.6
Ashanti	2026	24.9
Western	2811	34.5

### Burden of malaria on businesses

#### Case burden

**Fig. 1:**
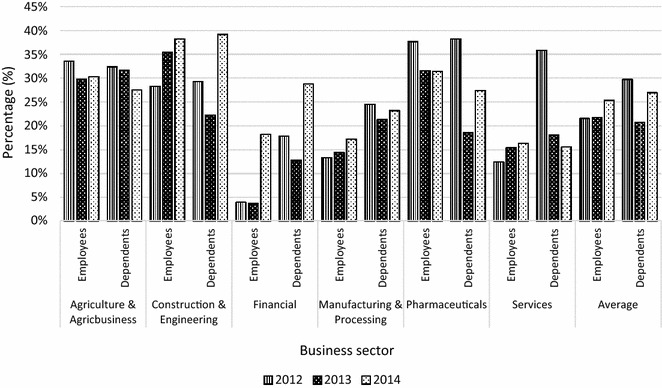
Percentage of employees and dependants who reported malaria

Over the three-year period, the average reported cases of malaria for all sectors was 22–25 % among employees and 21–30 % among dependants. The lowest burden was recorded in the financial sector i.e. 4–18 % among employees and 13–17 % among dependants. The highest burdens were 38 % among employees in the construction and engineering (2014) and pharmaceutical sectors (2012) and 39 % among dependants in the construction and engineering sector (2014). Figure 1 further shows that for 2014, the reported case burdens among employees was 38 % (construction and engineering), 31 % (pharmaceuticals), 30 % (agriculture and agribusiness), 18 % (financial), 17 % (manufacturing and processing) and 16 % (services). On average, 2014 saw the highest burden among employees. Again, the burden among dependants was higher than employees for services, manufacturing and processing, and financial sectors whereas the reverse holds for construction and engineering and pharmaceuticals sectors. For the agriculture and agribusiness sector, the average burden was the same for employees and dependants.

#### Cost burden of malaria

Table 2 shows that the total cost of malaria to businesses within the study sample for 2012–2014 was US$288,033.61, about 93 % of which was direct cost and about 7 % indirect cost.

**Table 2:** Annual economic cost (US$) of malaria to businesses

Type of cost	2012	2013	2014	Total	Cost profile (%)
Direct	99,406.85	96,429.05	73,436.74	269,272.64	93.4
Indirect	6265.12	6851.74	5644.11	18,760.97	6.6
Total	105,671.97	103,280.80	79,080.84	288,033.61	100.00

The estimated economic cost of malaria to businesses in Ghana in 2014 was US$6,588,729.09 (Table 3). Of this total, direct cost constituted 90 % and indirect cost constituted 10 %. Table 3 further shows that the economic burden was highest for businesses in the services sector (32 %), followed by businesses in the construction and engineering sector (about 22 %), financial sector (about 21 %) and lowest for businesses in the manufacturing and processing sector (about 2 %).

**Table 3:** Estimated costs (US$) of malaria to businesses by sector, 2014

Sectors	No. of businesses	Average cost (US$)		Total cost (US$)			Cost profile
		Direct	Indirect	Direct	Indirect	Total	
Agriculture and agribusiness	291	1906.60	69.85	554,819.29	20,325.11	575,144.41	8.7
Construction and engineering	453	3092.32	170.58	1,400,820.19	77,271.55	1,478,091.74	22.4
Financial	847	1138.94	519.37	964,681.41	439,903.06	1,404,584.47	21.3
Man. and processing	254	553.48	57.46	140,583.95	14,593.66	155,177.61	2.4
Pharmaceuticals	456	1762.21	145.16	803,569.84	66,190.94	869,760.77	13.2
Services	3527	585.25	11.85	2,064,186.44	41,783.66	2,105,970.10	32.0
Total	5828	–	–	5,928,661.12	660,067.97	6,588,729.09	100.0

#### Corporate expenditure on malaria treatment

On average between 2012 and 2014, the businesses in the sample spent 0.5 % of their gross returns on the treatment of malaria in their employees and dependants. The sectoral spending ranges between 0.1 % in manufacturing and processing to 0.9 % in services. In terms of annual averages, 2012 saw the highest average corporate spending on malaria treatment (0.6 %), driven by the construction and engineering and services sectors, which spent 1.2 % each of their gross returns on malaria treatment. Overall, businesses in the manufacturing sector spent the lowest on malaria treatment in 2012 i.e. 0.05 % (Table 4).

**Table 4:** Malaria treatment as percentage of gross returns on businesses

Sector	2012	2013	2014	Sector average
Agriculture and agribusiness	0.2	0.1	0.2	0.2
Construction and engineering	1.2	0.4	0.8	0.8
Financial	0.1	0.3	0.2	0.2
Manufacturing and processing	0.05	0.1	0.1	0.1
Pharmaceuticals	0.7	0.9	0.6	0.7
Services	1.2	0.9	0.5	0.9
Average	0.6	0.5	0.4	0.5

#### Corporate expenditure on malaria prevention activities

Table 5 shows that on average between 2012 and 2014, the businesses in the sample spent 0.3 % of their gross returns on malaria prevention activities. The sectoral spending ranges between 0.1 % for services, construction and engineering and agriculture and agribusiness and 0.6 % in manufacturing and processing. In terms of annual averages all sectors spent annual average of 0.3 % for each year. Overall, businesses in the service sector spent the lowest of 0.04 % in 2012 on malaria prevention activities.

**Table 5:** Expenditure on malaria prevention activities as percentage of gross returns

Sector	2012	2013	2014	Sector average
Agriculture and agribusiness	0.1	0.1	0.1	0.1
Construction and engineering	0.1	0.1	0.1	0.1
Financial	0.4	0.6	0.3	0.4
Manufacturing and processing	0.5	0.5	0.8	0.6
Pharmaceuticals	0.5	0.8	0.2	0.5
Services	0.0	0.1	0.1	0.1
Average	0.3	0.3	0.3	0.3

#### Corporate expenditure on other health-related corporate social responsibilities

On average between 2012 and 2014, the businesses spent 0.5 % of their gross returns on health related corporate social responsibility (CSR). The sectoral spending ranges between 0.1 % in pharmaceuticals, agriculture and agribusiness and construction and engineering and processing to 1.3 % in services. In terms of annual averages, 2014 saw the highest average corporate spending of 0.8 %, driven by the services, financial, manufacturing and processing who spent more than 1 %. Overall, businesses in the pharmaceutical sector spent the lowest of 0.02 % in 2014 on other health-related CSR (Table 6).

**Table 6:** Expenditure on other health-related corporate social responsibilities

Sector	2012	2013	2014	Sector average
Agriculture and agribusiness	0.1	0.1	0.2	0.1
Construction and engineering	N/A	0.2	0.1	0.1
Financial	0.3	0.6	1.3	0.7
Manufacturing and processing	1.0	0.5	1.0	0.8
Pharmaceuticals	0.1	0.2	0.0	0.1
Services	0.3	1.6	2.0	1.3
Average	0.3	0.5	0.8	0.5

**Fig. 2:**
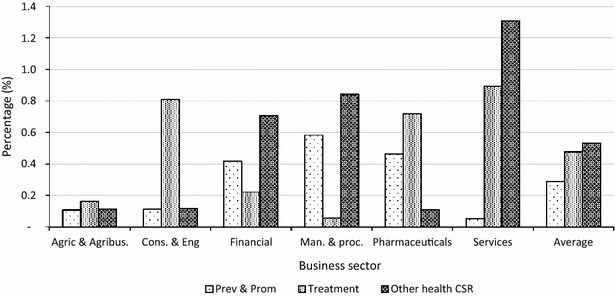
Proportion of spending on malaria treatment, prevention and other CSR

Figure 2 shows that the manufacturing and processing and financial sectors spent more on malaria prevention activities between 2012 and 2014 compared to malaria treatment. Also services, manufacturing and processing and financial sectors reported higher expenditure on other health-related CSRs than malaria prevention and treatment. Furthermore, agriculture and agribusiness, construction and engineering, and pharmaceutical sectors expended more of corporate returns on malaria treatment than malaria prevention or other health-related CSR (Fig. 2).

### Productive days lost by businesses due to malaria

**Fig. 3:**
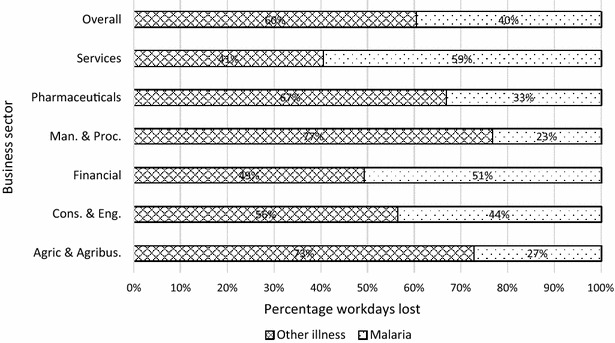
Proportion of productivity days lost due to malaria by sector

Over the period 2012–2014, the businesses in the study sample (i.e. 62 businesses across the three regions) lost a total of 3913 workdays due to malaria (Table 7). The lowest days lost was recorded by manufacturing and processing (54 days) and highest by services (1620 days). The agriculture and agribusiness and services sectors together accounted for 77 % of the total workdays lost. The highest number of work days lost was recorded in 2014 (i.e. 1530 days). Table 8 presents the workdays lost by staff category. Table 8 shows that 73 % of the total workdays lost was recorded in junior staff followed by 17 % in senior staff and 10 % in management. Figure 3 shows that overall, malaria accounted for 40 % of productive days lost due to illnesses in all sectors. This ranges from 23 % in manufacturing and processing to 59 % in services.

**Table 7:** Annual productivity days lost due to malaria

Sector	2012	2013	2014	Total	Period average
Agriculture and agribusiness	339	448	607	1394	35.6
Construction and engineering	151	211	209	571	14.6
Financial	35	22	93	150	3.8
Manufacturing and processing	23	19	12	54	1.4
Pharmaceuticals	41	39	44	124	3.2
Services	582	473	565	1620	41.4
Total	1171	1212	1530	3913	100.0

**Table 8:** Productive days lost by staff category

Staff category	2012	2013	2014	Total	Period average
Junior staff	873	888	1092	2853	72.9
Senior staff	203	228	232	663	16.9
Management	95	96	206	397	10.1
Total	1171	1212	1530	3913	100.0

### Other effects/burden of malaria

#### Effect of malaria on business operations

**Fig. 4:**
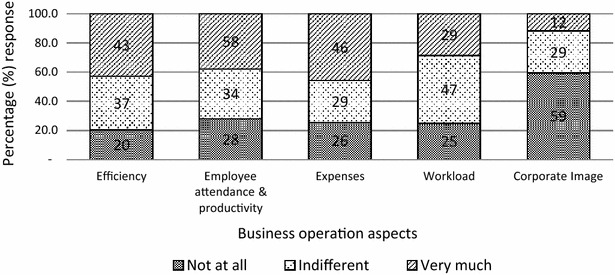
Effect of malaria on business operations

Figure 4 shows that a higher proportion of business leaders from the study sample reported that malaria affected efficiency (43 %), employee attendance and productivity (38 %) and expenses (46 %). About 59 % further reported that malaria did not affect their corporate image while about 47 % reported being indifferent to the effect of malaria on workload.

Business leaders (Chief Executive Officer/Managing Directors, Operations managers and Human Resource managers) representing the sample of 62 businesses expressed the view that employee absenteeism due to malaria affected their businesses through reduced workforce productivity, reduced business outputs and increased operation cost (through high medical expenses). They also indicated that worker absenteeism distorts their timelines and targets/production chain.

Asked whether it is worthwhile for government to increase investments in malaria control, about 98 % of the 130 business leaders responded in the affirmative and explained that such increased investments would reduce morbidity and mortality, boost business outputs and improve economic growth. Asked whether it is worthwhile for their specific businesses to invest in malaria control activities, about 93 % of the business leaders responded in the affirmative.

## Discussion

The study findings show that during the period 2012–2014, the total cost of malaria to businesses in the study sample was US$288,033.61, 93 % of which constituted direct costs. Extended to the total number of businesses in the country, the estimated cost of malaria to businesses in Ghana in 2014 was about US$6.58 million, 90 % of which were direct costs. Estimates of the economic cost of malaria to businesses in developing countries, especially sub-Saharan Africa, are rare. Studies have estimated the economic cost of malaria on the Ghanaian economy—US$50.05 million in 2002 costs by Asante and Asenso-Okyere [17]; US$66.9 million in 2009 costs by Sicuri et al. [19]; reductions in malaria would increase economic growth by 0.07 % bringing in additional annual incomes of US$279–US$298 million (2007 costs) over 30 years [23]. However, most of these studies do not account for the cost of malaria to businesses in their analyses mainly because of data limitations, i.e. difficult to find malaria-specific morbidity and mortality data at firm level but also most firms are normally either unwilling or uncomfortable releasing data on business output and returns for such purposes.

Further, for the period 2012–2014, a total of 3913 workdays were lost due to malaria in the 62 firms that constituted the study sample, implying that on average, each business lost about a month’s productivity per year due to malaria. About 73 % of the total workdays lost represented productive losses in junior staff. This is significant because more often, junior staff are directly linked to production levels in many firms. Thus, this significant burden on them directly impacts the output of firms. In terms of sectoral analysis, the services sector constituted about 41 % of the total workdays lost, followed by agriculture and agribusiness sector with 36 %. According to the Ghana Statistical Service [24], services and agriculture were the first and third largest sectors in the Ghanaian economy in 2013, contributing 49.5 and 22.0 %, respectively, to GDP. Thus, the loss of more than 1300 workdays in each sector (i.e. in only 62 businesses) should be a source of worry to not only government but also businesses.

Furthermore, this study found that during the period 2012–2014, business spent about 0.5 % of annual corporate returns on treatment of malaria in employees and their dependants, with the services sector spending about 1 % and manufacturing sector spending about 0.1 % annually. These estimates may seem insignificant. However, for large businesses, these translate into significant costs to business, thereby raising overall cost of production and potentially reducing profits. Using this analogy, businesses could save more by raising their spending on prevention of malaria (which stood at 0.3 % per annum between 2012 and 2014) as reduction in malaria due to such efforts would lead to reductions in medical expenses and overall cost of production and, consequently lead to improved returns. Such efforts would appear costly over the short term. However, over a longer term, these would lead to appreciable improvements in business profits.

It is important to note that the study finds that the services sector which spent the least proportion of gross returns on malaria prevention activities spent the largest on malaria treatment in employees and their dependants. The reverse holds for the manufacturing and processing sector—which spent the largest of 0.6 % of gross returns on malaria prevention over the study period and spent the least on malaria treatment in employees and their dependants. The current study did not attempt to model the relationship between business spending on malaria prevention and treatment. However, it follows that if a business spends more on preventing malaria at the workplace and within the communities they operate, its employees and dependants would have reduced burden of malaria. It would be interesting for further studies to map out the specific activities that businesses undertake with respect to malaria prevention and also other health-related CSRs.

The survey of leaders of the businesses in the study sample indicates that businesses have a clear understanding malaria affects their businesses in various ways. They indicated that employee absenteeism due to malaria (in employees but also in their dependants) affected their businesses through reduced workforce productivity, reduced business outputs and increased operation cost (through high medical expenses). They also indicated that worker absenteeism distorts their timelines and targets/production chain. Beyond worker absenteeism, business leaders further indicated that malaria had negative impacts on the efficiency of their businesses and on their overall expenses through increased medical bills.

The overwhelming majority (98 %) of business leaders expressed the opinion that there is the need for increased government investments in malaria control. Such increased investments, they believe, would improve health and social wellbeing through reduced morbidity and mortality, which would then improve participation of households in income generating activities that could reduce poverty, but also more school days that could increase education outcomes in the longer term. On businesses, business leaders indicated that increased government investments in malaria control would reduce worker absenteeism and improve workforce productivity. A combined effect of improved productivity and reduced production cost (as a result of savings in medical expenses, etc.) would improve business profits, which would have two effects—increase corporate tax to government and attract investors into the sector due to profitability, which could further boost corporate taxes, thereby enhancing government revenue. Improved business output and government revenue would lead to improved economic outlook and enhance economic growth.

These findings are consistent with the views of business leaders worldwide; 22 % of 8000 business leaders worldwide reported that malaria negatively impacted their businesses; 72 % of respondents from SSA reported that malaria affected their businesses, with 40 % reporting that malaria had “serious detriments” on their businesses [10, 11]; out of the 119 respondents from Ghana in the same survey, 30 % reported that malaria impacts productivity; respondents concurred that reduced malaria burden would improve productivity, reduce costs of production, improve sales and widen opportunities for marketing [10].

The study further finds that the overwhelming majority (93 %) of the business leaders surveyed responded that it is worthwhile for their businesses to invest in malaria control. This finding is important and forms the basis for the involvement of the private sector in malaria control. Private sector involvement in malaria control may come in different forms; businesses may be engaged to contribute to a national pool of funds to be used in scaling up current interventions; groups of businesses located within same communities may be engaged to support malaria control activities within their catchment communities i.e. financial support for interventions, such as indoor residual spraying and insecticide-treated bed net distribution, within their communities. Businesses may also be involved in malaria control through education and campaigns (through production and distribution of information, education and communication materials) directed towards their staff and dependants, clients and their immediate communities. Research and development (R&D) are a crucial part of efforts toward malaria control and elimination. Therefore, businesses may be engaged to support R&D through support for research into specific aspects of malaria control and training.

It is important to note that a key limitation faced by the current study relates to the data from businesses; some large businesses were unwilling to be part of the study. For those who were part, many reported a lack of specific data on malaria morbidity and costs. Thus, for these businesses, we used the national estimates of proportion of malaria to estimate malaria morbidity (out of total morbidity) and costs. It is also important to note that the national estimates of costs only involved businesses registered with the Association of Ghana Industries and the Ghana Employers Association. However, there are businesses that may not be part of these two bodies, which were excluded from the analysis. Consequently, the estimates from this study are likely to be an underestimation of the true economic cost of malaria to businesses in Ghana. Further, the indirect cost (productivity losses due to worker absenteeism) was estimated using the national daily minimum wage because data on gross salaries of staff were not collected due to difficulties in getting these from businesses. It is possible that the use of minimum wage underestimates the actual indirect costs since many businesses pay their staff salaries higher than the national daily minimum wage.

In spite of these limitations, this study is an attempt at estimating the cost of malaria to businesses in Ghana. Further studies are needed to estimate the impact of malaria-specific corporate spending on the growth and returns of businesses. This would require time series data to effectively model such relationship. Thus, businesses would have to consistently keep records on disease-specific morbidity and expenditure associated with such morbidity.

## Conclusions

The current study has estimated the total cost of malaria to businesses in Ghana in 2014 to be US$6.58 million, 90 % of which were direct costs. Further, a total of 3913 workdays were lost due to malaria in firms in the study sample during the period 2012–2014, with an annual average of 1304. The study further estimates that businesses in the study sample spent an average of 0.5 % of the annual corporate returns on treatment of malaria in employees and their dependants, 0.3 % on malaria prevention, and 0.5 % on other health-related corporate social responsibilities. Business leaders affirmed that malaria affects their businesses’ efficiency, employee attendance and productivity and expenses. Finally, majority of business leaders expressed the need for increased government investments in malaria control and most of them concurred that it is worthwhile for their businesses to invest in malaria control. Thus, business leaders do acknowledge the effect of malaria morbidity on their operations and ultimately their income and workforce. This acknowledgement provides motivation for the private sector to be engaged in malaria control activities.

## Abbreviations

AGI: Association of Ghana Industries
CSR: corporate social responsibility
DFID: UK: Department for International Development
GEA: Ghana Employers Association
GHS: Ghana Health Service
GSS: Ghana Statistical Service
GDP: gross domestic product
NMCP: National Malaria Control Programme
R&D: Research and Development
SSA: Sub-Saharan Africa
WHO: World Health Organization
